# Influenza A Virus Infection of Human Primary Dendritic Cells Impairs Their Ability to Cross-Present Antigen to CD8 T Cells

**DOI:** 10.1371/journal.ppat.1002572

**Published:** 2012-03-08

**Authors:** Anna Smed-Sörensen, Cécile Chalouni, Bithi Chatterjee, Lillian Cohn, Peter Blattmann, Norihiro Nakamura, Lélia Delamarre, Ira Mellman

**Affiliations:** Genentech, South San Francisco, California, United States of America; Mount Sinai School of Medicine, United States of America

## Abstract

Influenza A virus (IAV) infection is normally controlled by adaptive immune responses initiated by dendritic cells (DCs). We investigated the consequences of IAV infection of human primary DCs on their ability to function as antigen-presenting cells. IAV was internalized by both myeloid DCs (mDCs) and plasmacytoid DCs but only mDCs supported viral replication. Although infected mDCs efficiently presented endogenous IAV antigens on MHC class II, this was not the case for presentation on MHC class I. Indeed, cross-presentation by uninfected cells of minute amounts of endocytosed, exogenous IAV was ∼300-fold more efficient than presentation of IAV antigens synthesized by infected cells and resulted in a statistically significant increase in expansion of IAV-specific CD8 T cells. Furthermore, IAV infection also impaired cross-presentation of other exogenous antigens, indicating that IAV infection broadly attenuates presentation on MHC class I molecules. Our results suggest that cross-presentation by uninfected mDCs is a preferred mechanism of antigen-presentation for the activation and expansion of CD8 T cells during IAV infection.

## Author Summary

Although the interactions between viruses and dendritic cells (DCs) have been studied for many years, surprisingly little is known on the functional relationship between infection and antigen presentation in primary human DCs. Here, we asked specifically whether Influenza A virus (IAV) infection of human primary plasmacytoid DCs and myeloid DCs (pDCs and mDCs, respectively) affected their ability to function as antigen-presenting cells and activate T cells specific to IAV or other antigens. Our data confirm that pDCs are poorly infected and also present IAV antigens poorly. mDCs, on the other hand, are readily susceptible to IAV infection and present IAV antigen to T cells. However, we found that MHC class I presentation by mDCs infected with IAV are ∼300-fold less efficient relative to what mDCs are capable of achieving by cross-presentation following the endocytosis of only a very few non-infectious virions. Importantly, IAV infection of mDCs not only reduces the efficiency of IAV presentation but also reduces their ability to cross-present antigens from other viruses encountered subsequently. The reduced overall antigen processing capacity of mDCs describes a mechanism that may contribute to the suppression of immunity to secondary pathogens that appear during the course of IAV infection.

## Introduction

Influenza A virus (IAV) infection is one of the oldest and most common diseases known to mankind, estimated to cause 500,000 deaths per year, primarily in infants and elderly [1]. In healthy humans, IAV infection typically causes brief but often severe illness. Normally, IAV infection is confined to the airways where the virus replicates in respiratory epithelial cells [2]. Rapidly, alveolar macrophages produce pro-inflammatory cytokines and chemokines, which promote infiltration of peripheral blood leukocytes to the site of infection [3]. While influx of neutrophils and secretion of cytokines and chemokines in the lung is a fundamental defense during the initial stage of infection, the resulting “cytokine storm” may also contribute to pathogenesis [4]. However, control and clearance of IAV infection depend on pathogen-specific adaptive immune responses [5].

The initiation of adaptive immunity relies on dendritic cells (DCs), professional antigen-presenting cells (APCs) with the capacity to activate naïve T cells [6]. The two major subsets of human DCs, myeloid and plasmacytoid DCs (mDCs and pDCs, respectively) both have antigen-presenting capacity although mDCs are generally considered to be superior. pDCs are of central importance in virus infections since they respond rapidly to viruses and secrete high levels of anti-viral type I interferons [7]. DCs reside in the epithelia of the upper respiratory tract, the site of entry for IAV, and are also rapidly mobilized to this site following inhalation of microbial agents [8]–[9]. Since there is little evidence of viral replication in lymphoid tissue, the main source of IAV antigen is thought to be DCs that exit the respiratory tract and travel to lymphoid tissue where immune responses are initiated [10]–[11].

During acute viral infections, activation and expansion of antigen-specific CD8 T cells are crucial for control and clearance of infection [5], [12]–[13]. In general, MHC class I molecules (MHCI) present peptides derived from endogenously synthesized proteins. Viruses that replicate in DCs can therefore be detected by the immune system by direct presentation of viral antigens. Since not all viruses infect DCs, antigen-presentation by uninfected DCs is thought to occur via cross-presentation, a poorly understood process unique to DCs where exogenous antigen is loaded on MHCI in the ER or possibly other intracellular compartments [14]. Mice lacking CD8α+ DCs are deficient in their capacity to mount an anti-viral immune response [15] suggesting that cross-presentation is crucial for a CD8 T cell response against viruses. On the other hand, it could also suggest that direct presentation by virus infected CD8α+ DCs is required for CD8 T cell responses. The relative contributions of direct versus cross-presentation for the induction of anti-viral CD8 T cell responses have been a topic of discussion for several years [16]–[19], and have been compared in mouse models [19]–[22]. However, the efficiency of direct versus cross-presentation of IAV and the potential IAV-mediated suppression of antigen-presentation in human DCs remains an unresolved topic.

IAV infection often predisposes individuals to secondary infections, usually bacterial, with higher lethal outcomes than either infection alone, suggesting that the initial infection affects the host's ability to respond to a second pathogen. While the connections between viral and bacterial infections have been known for decades [23], the mechanism(s) and modes of interaction contributing to these effects are poorly understood. IAV infection clearly suppresses innate immune responses [24]–[27], but the extent to which adaptive immune responses are also affected, and why, remains unclear.

While IAV infection has been studied extensively in animal models, relatively little is known about how IAV infection affects the function of human DC subsets due in part to their limited availability, making such experiments challenging. In mice, mDCs, rather than pDCs, appear to be responsible for presenting IAV antigen to CD8 T cells for the priming of anti-IAV immune responses [28]–[31]. In humans, it is much less clear what subset(s) of DCs are important in antigen-presentation during IAV infection. Human DCs infected with infectious IAV or exposed to inactivated IAV can activate IAV-specific T cells [32]–[36], however, it remains unclear if or how IAV infection of human DCs affects their function. Here, we investigated the consequences of IAV infection on the ability of DCs to present IAV antigen, or other antigens, to autologous T cells using relevant subsets of primary human DCs.

## Results

### mDCs but not pDCs are susceptible to influenza A virus infection

**Figure 1:**
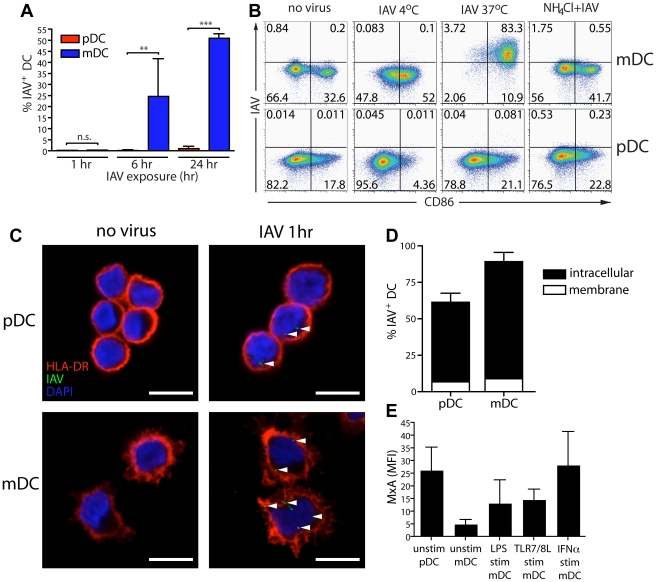
mDCs but not pDCs are susceptible to IAV infection. (**A**) pDCs (red) and mDCs (blue) were continuously exposed to IAV for 1, 6 or 24 hr and the frequency of IAV+ DCs was measured by flow cytometry using a rabbit polyclonal antibody raised against IAV/X31. Graph shows mean±SD percent of IAV+ CD123+ CD14− pDCs and CD11c+ CD14− mDCs (n = 7). Differences between IAV infection of mDCs and pDCs were assessed using paired t test: n.s. no significant difference, ** p<0.01, *** p<0.001. (**B**) DCs were treated with NH_4_Cl, then exposed to IAV for 24 hr at 4°C or 37°C. The frequency of IAV+ DCs and level of CD86 expression was determined by flow cytometry. One representative experiment of six is shown. Dot plots show live DCs and numbers in each quadrant depict the frequency of positive DCs. (**C**) DCs were exposed to IAV for 1 hr, washed 3 times to remove free virus and allowed to adhere to coverslips. Surface HLA-DR (red) was labeled before fixation to visualize the plasma membrane. After permeabilization, virus was stained using an anti-IAV antibody (green) and the nucleus was stained using DAPI (blue). The entire volume of each cell was analyzed using confocal microscopy (100× 1.47NA oil objective, 6× digital zoom) and one single optical slice through the center of the cell is shown with arrowheads pointing to virus structures. Scale bar 10 µm. (**D**) The frequency of IAV+ DCs after 1 hr of virus exposure was determined by analyzing entire z-stacks of DCs and counting the number of cells that had virus associated with them, either on the membrane (white) or intracellularly (black). The graph shows average frequency of IAV+ DCs±SD, with 100–300 DCs analyzed per donor and condition (n = 4). (**E**) DCs were treated with LPS, TLR7/8L, IFNα or nothing for 24 hr and the MxA expression was determined by flow cytometry using intracellular staining with a monoclonal anti-MxA antibody. Graph shows MFI±SD of MxA with isotype control subtracted (n = 3).

pDCs are known to be more resistant to the cytopathic effect of IAV than mDCs, suggesting that pDCs are resistant to infection [36], [37]. To extend this observation and to determine any possible consequences for antigen-presentation, primary human mDCs and pDCs were exposed to IAV and the frequency of IAV+ DCs was analyzed. While the frequency of IAV+ mDCs increased over time, infection in pDCs remained undetectable (Figure 1A). The IAV+ mDCs reflected the production of newly synthesized viral proteins rather than enhanced virion uptake since adding the virus at 4°C, or blocking virus endosomal egress with NH_4_Cl, inhibited the appearance of IAV+ DCs (Figure 1B). Interestingly, infectious IAV was not detected in the supernatant even after 24 hr, indicating that despite high viral protein production, mDCs did not support generation of infectious particles (Figure S1A). These observations were confirmed for several IAV strains (Figure S1B).

To assess whether the lack of infection in pDCs reflected poor endocytosis of virus, human mDCs and pDCs were exposed to IAV and analyzed by confocal microscopy. After 1 hr, the majority of both DC subsets displayed internalized virus (Figures 1C–D), suggesting that other factors blocked pDC infection, such as the pDCs' constitutive expression of the interferon-inducible antiviral protein MxA (Figures 1E and S2) [37]. It has previously been shown that expression of MxA renders cells resistant to IAV infection [38]. We were unable to knockdown MxA in pDCs using siRNA while maintaining pDC viability (data not shown). Still, the constitutive high expression of MxA in pDCs suggests that this protein could aid in the observed resistance to IAV infection, despite efficient IAV internalization by pDCs.

**Figure 2:**
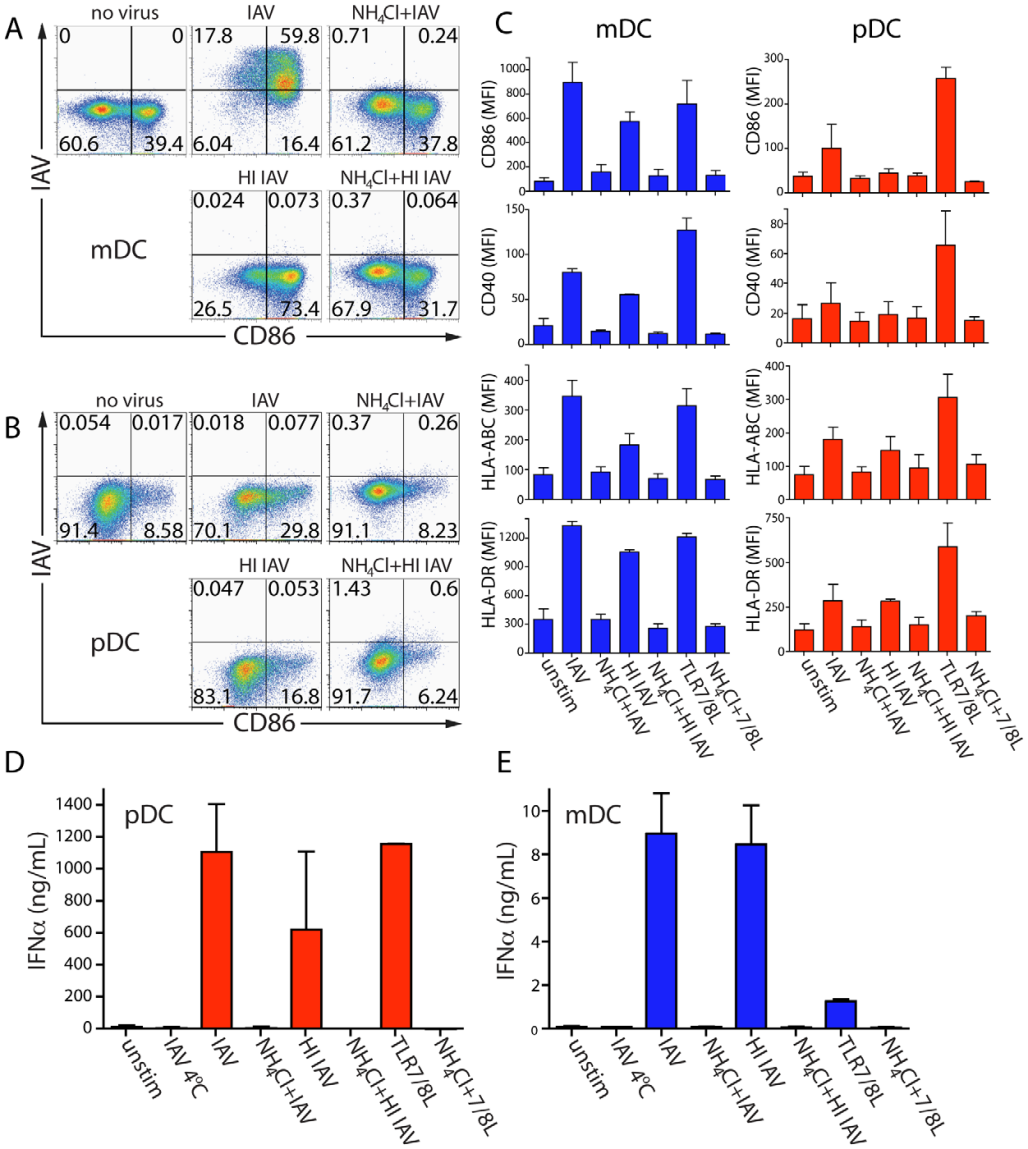
Both infectious IAV and HI IAV induce maturation and cytokine secretion in mDCs and pDCs in a pH-dependent manner. mDCs (**A**) or pDCs (**B**) were exposed to infectious IAV or HI IAV in the absence or presence of NH_4_Cl for 24 hr. The frequency of IAV+ DCs and their CD86 expression was determined. Dot plots show live DCs and numbers in each quadrant depict the frequency of positive DCs. One representative donor of eight is shown. (**C**) The surface expression of CD86, CD40, MHCI (HLA-ABC) and MHCII (HLA-DR) on mDCs (blue) and pDCs (red) was determined after 24 hr of exposure to infectious IAV, HI IAV or TLR7/8L in the absence or presence of NH_4_Cl. Bar graphs show MFI±SD (n = 3). The levels of secreted IFNα from supernatants of pDCs (**D**) or mDCs (**E**) after 24 hr were determined by ELISA. The graphs show mean±SD (n = 9).

pDCs, like mDCs, were nevertheless found to respond to the presence of IAV even in the absence of the synthesis of virus-encoded proteins. This was illustrated by comparing the ability of infectious IAV and non-infectious heat-inactivated (HI) IAV to trigger DC maturation. Both replicating and HI IAV were internalized equivalently. In addition, both could fuse with the endosomal membrane at low pH, as indicated by agglutination and acid-dependent lysis of chicken red blood cells (data not shown). As expected, HI IAV did not infect DCs, and infection of mDCs by replication-competent IAV was blocked by NH_4_Cl (Figures 2A–B). Yet, both pDCs and mDCs upregulated MHCI and MHCII in response to infectious and HI IAV (Figure 2C). Furthermore, pDCs responded by secreting large amounts of IFNα (Figure 2D). This was true for several IAV strains (Figure S3). mDCs also secreted IFNα in response to IAV, although the levels were 100–1000 fold lower than for pDCs (Figure 2E). pDCs recognize IAV via TLR [39] while most cells respond to single stranded RNA viruses via the RIG-I pathway [40]. Human primary mDCs express TLR7 and TLR8 that recognize single-stranded RNA. The virus-related TLRs can be stimulated by inactivated viruses, however, the barely detectable amount of IFNα secreted by mDCs in response to TLR7/8L suggests that this was a consequence of signaling via cytoplasmic receptors rather than via TLRs (Figure 2E). In addition, pDCs secreted TNFα, IL-6 and MIP-1α in response to IAV, while mDCs required stimulation with purified TLR7/8 ligand (TLR7/8L) to secrete significant amounts of cytokines and chemokines (Figure S4). Thus, IAV enters both mDCs and pDCs and triggers their maturation, but only mDCs support viral protein synthesis.

### mDCs are superior at MHCI restricted antigen-presentation of IAV compared to pDCs

**Figure 3:**
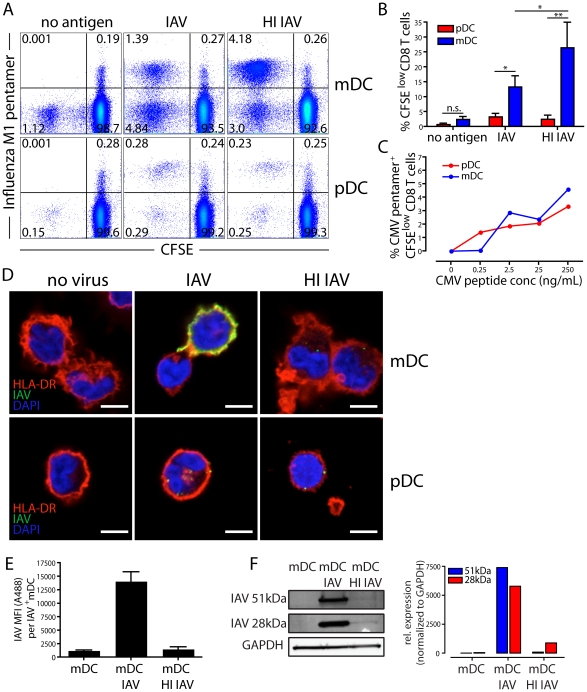
Cross-presentation is more efficient than direct presentation of IAV. (**A**) DCs were exposed to infectious IAV, HI IAV or left untreated for 4 hr, washed and co-cultured with autologous CFSE labeled CD8 T cells at 1∶30 DC∶T cell ratio. After 10 days of co-culture, cells were harvested and stained with an HLA-A2 Influenza A M1 pentamer to detect M1-specific CD8 T cells by flow cytometry. T cell proliferation was detected by CFSE dilution. Dot plots show live CD8 T cells and numbers indicate frequency of positive CD8 T cells in each quadrant. One representative donor of four is shown. (**B**) Frequency of live CFSE^low^ CD8 T cells co-cultured with mDCs (blue) and pDCs (red) for 10 days, as described in (A), as a measurement of the total CD8 T cell response to IAV. Graph shows mean±SD from four donors. Differences were assessed using paired t test: n.s. no significant difference, * p<0.05, ** p<0.01. (**C**) DCs were exposed to increasing doses of pre-processed CMV pp65 peptide for 3 hr, washed and co-cultured with autologous CFSE-labeled CD8 T cells. After 10 days of co-culture, cells were harvested and stained with a CMV pp65 pentamer to detect CMV pp65-specific CD8 T cells by flow cytometry. The graph shows frequency of CMV pentamer+ CFSE^low^ CD8 T cells after co-culture with mDCs (blue) or pDCs (red) from one representative donor of five. (**D**) After 4 hr of exposure to infectious IAV or HI IAV, the expression of Influenza A protein was assessed using an anti-IAV polyclonal antibody (green) in HLA-DR (red) expressing MDCs and PDCs by confocal microscopy. Nuclei were stained using DAPI (blue). Scale bar 5 µm. (**E**) mDCs were exposed to infectious IAV or HI IAV for 8 hr and IAV+ mDCs were measured by MFI. The graph represents average MFI ± SD (n = 3) where 50–100 mDCs were analyzed for each donor. (**F**) mDCs were exposed to infectious IAV or HI IAV for 8 hr. Cells were lysed and analyzed by Western blot for viral protein content using a rabbit anti-IAV polyclonal antibody. The relative expression of IAV HA (51 kDa) and IAV M1 (28 kDa) was determined using ImageJ after normalizing the samples to GAPDH.

To determine if infected human mDCs and pDCs could present antigen to and activate IAV-specific CD8 T cells, we exposed DCs from HLA-A2+ donors to either infectious IAV or non-infectious HI IAV and co-cultured them with autologous CFSE-labeled CD8 T cells. After 10 days, the frequency of memory CD8 T cells specific to the immunodominant influenza M1 (58–66) epitope was determined and the overall CD8 T cell response assessed by CFSE dilution. While both mDCs and pDCs could expand IAV-specific CD8 T cells, mDCs were superior (Figures 3A–B). This difference likely reflected different capacities for antigen-processing since both subsets presented pre-processed peptide (that does not require cellular processing) similarly (Figure 3C). IAV presentation to CD8 T cells by pDCs was the same for both infectious and non-infectious virus, strongly suggesting that pDCs were only capable of cross-presenting IAV antigens, albeit inefficiently from virions internalized by endocytosis.

Presentation on MHCI is a hallmark of viral immunity since infected cells express virus-derived peptides recognized for elimination by cytotoxic CD8 T cells. In DCs, it is unclear if the generation of CD8 T cell responses reflects the formation of peptide-MHCI complexes from endogenously synthesized viral proteins or the cross-presentation of antigens from exogenous sources (e.g. internalized virions or infected cells) [41]. Indeed, DCs are well known to have an enhanced capacity for cross-presentation, which requires that internalized antigens exit the endosomal pathway for peptide cleavage in the cytosol and subsequent loading onto MHCI molecules in the ER or elsewhere [14]. Although HI IAV cannot infect DCs, it retains the capacity to fuse with the endosomal membrane [32] thus providing an intrinsic capacity to reach the cytosol, which is possibly the rate-limiting step in the cross-presentation [42].

Strikingly, mDCs exposed to HI IAV induced a statistically significant, two-fold more effective expansion of IAV-specific CD8 T cells than mDCs infected with IAV (Figures 3A–B). This was surprising because it is generally assumed that presentation of peptides from endogenously synthesized proteins is more efficient than cross-presentation [19]. In addition, IAV infected DCs expressed far greater amounts of IAV proteins than DCs exposed to HI IAV. This was readily apparent (Figures 3D and 2A); although infected cells stained heavily for IAV proteins, cells exposed to HI IAV contained only 4–5 virions (defined as IAV+ puncta) per cell (Figure S5). We determined the mean fluorescence intensity (MFI) of the IAV staining in IAV+ mDCs and found that mDCs infected with infectious IAV displayed 10-fold more IAV staining than mDCs exposed to HI IAV (Figure 3E). To more accurately determine the relative content of IAV proteins, we next analyzed cell lysates of mDCs exposed to infectious or HI IAV by Western blot. Due to continued synthesis in the infected cells, and continued degradation of virions in the HI IAV exposed cells, a quantitative comparison was no more than an estimate. Yet, influenza proteins were present at 100–1000 fold higher amounts in infected cells as compared to cells that had internalized HI IAV (Figure 3F). The 28 kDa band most likely corresponds to the M1 protein, ∼3000 copies of which are contained within each virion, or 15,000 copies per cell after endocytosis of 5 virions. Assuming all of the HI IAV fuse with the endosome membrane releasing all of the incoming M1 into the cytosol, we estimate that the uninfected DCs are at least 300-fold more efficient at stimulating M1-specific CD8 T cells than infected DCs: i.e. despite vastly greater amounts of cytosolic M1, IAV infected DCs process and present M1-derived peptides to CD8 T cells less well. This difference in antigen processing and presentation translates into the observed difference in frequency of proliferating IAV-specific CD8 T cells depicted in Figure 3B.

On the other hand, pDCs exposed to either infectious IAV or HI IAV were comparable in their ability to expand IAV-specific CD8 T cells (Figures 3A–B). Comparing presentation of HI IAV by mDC and pDCs, pDCs were 10–20 fold less effective at cross-presentation than mDCs (Figures 3A–B). This difference was consistent over a range of IAV concentrations and DC∶T cell ratios (Figure S6).

**Figure 4:**
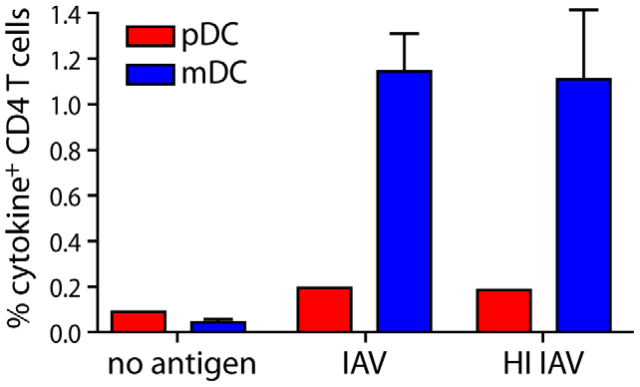
DCs exposed to infectious IAV and HI IAV are similar in MHCII restricted antigen-presentation. pDCs (red) and mDCs (blue) were exposed to infectious IAV, HI IAV or left untreated for 4 hours, washed and co-cultured with autologous CD4 T cells at a 1∶30 DC∶T cell ratio. After 1 hr of co-culture brefeldin A was added. After another 16 hr of co-culture, cells were harvested and stained with antibodies to detect cytokine-producing CD4 T cells and analyzed by flow cytometry. Graph shows mean±SD percent of live, IFNα, TNFα, IL-2+ CD4 T cells (n = 3).

Contrary to CD8 T cell responses, CD4 T cells responded comparably to mDCs exposed to either infectious or HI IAV. As with presentation on MHCI, mDCs were superior to pDCs for MHCII-restricted presentation (Figure 4). Thus, IAV infection did not diminish the efficiency of presentation to CD4 T cells, showing that the effect on the MHCI pathway was selective.

### IAV infected mDCs have an impaired capacity to cross-present other viral antigens

IAV infection often predisposes individuals to secondary infections, suggesting that infection history affects the ability to mount adaptive responses to new pathogens. Therefore, we investigated if uninfected and IAV infected DCs were comparable in their ability to present a second antigen to CD8 T cells and support their activation and expansion. The most common secondary infection in IAV infected individuals is *Streptococcus pneumoniae*. However, in the absence of tools to look at potential T cell responses against *S. pneumoniae*, we made use of existing immunodominant CD8 T cell memory responses against EBV and CMV in HLA-A2+ donors as model antigens. Since pDCs were not susceptible to IAV infection, we focused on mDCs.

**Figure 5:**
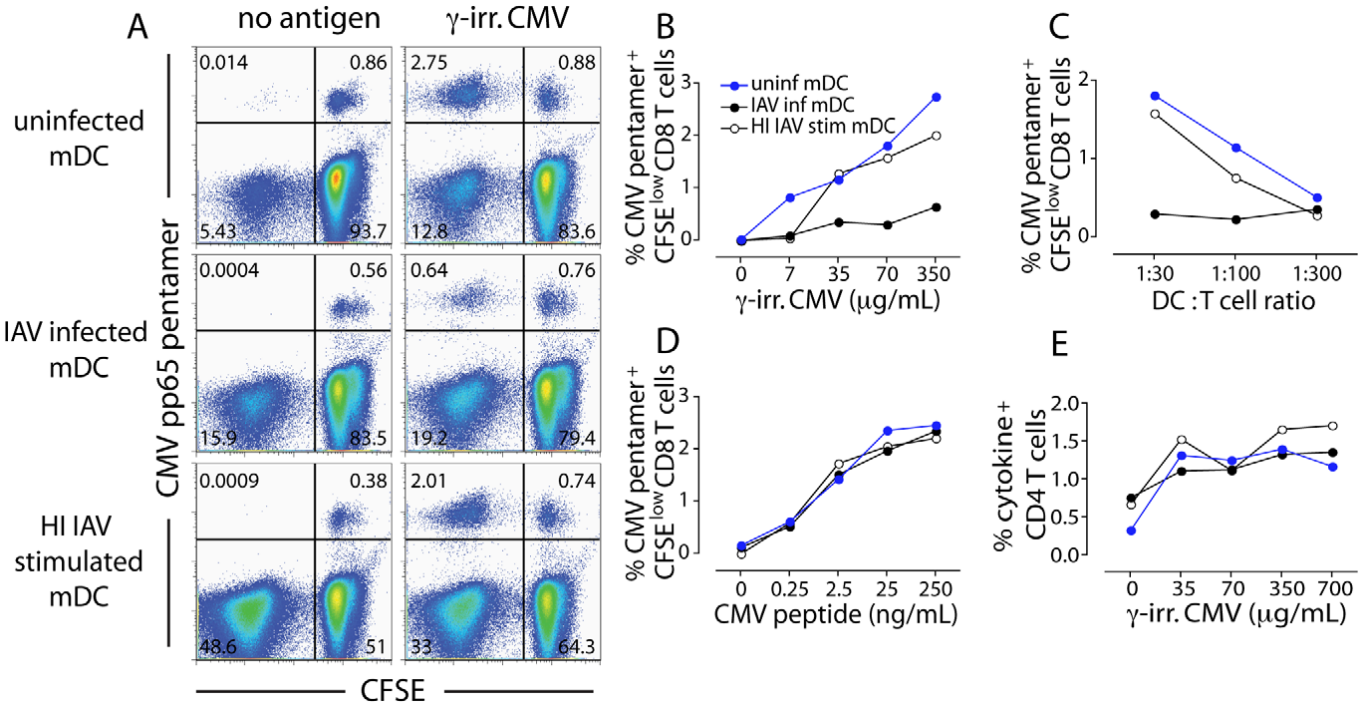
Uninfected mDCs are superior at cross-presentation than IAV infected mDCs. (**A**) mDCs were exposed to infectious IAV, HI IAV or left untreated for 4 hr and washed, then exposed to whole, γ-irradiated CMV for an additional 3 hr, washed and co-cultured with autologous CFSE-labeled CD8 T cells. After 10 days of co-culture, cells were harvested and stained with an HLA-A2 CMV pp65 pentamer to detect CMV pp65-specific CD8 T cells and analyzed by flow cytometry. Dot plots show live CD8 T cells and numbers indicate frequency of positive CD8 T cells. One representative experiment of five is shown. (**B**) Frequency of live CMV pentamer+ CFSE^low^ CD8 T cells co-cultured with uninfected mDCs (blue), IAV infected mDCs (black) or HI IAV stimulated mDCs (white) for 10 days, as described in (A) where mDCs were fed increasing doses of whole, γ-irradiated CMV before co-culture with CD8 T cells. (**C**) Frequency of live CMV pentamer+ CFSE^low^ CD8 T cells co-cultured with uninfected mDCs (blue), IAV infected mDCs (black) or HI IAV stimulated mDCs (white) for 10 days, as described in (A) after co-culture at different DC∶CD8 T cells ratios. (**D**) Uninfected mDCs (blue), IAV infected mDCs (black) or HI IAV stimulated mDCs (white) were exposed to increasing doses of pre-processed CMV pp65 peptide for 3 hr, washed and co-cultured with autologous CFSE-labeled CD8 T cells for 10 days. The graph shows frequency of live CMV pentamer+ CFSE^low^ CD8 T. One representative experiment of five is shown. (**E**) mDCs were exposed to infectious IAV, HI IAV or left untreated for 4 hr, washed to remove non-cell associated virus and exposed to 100 µL whole, γ-irradiated CMV for an additional 3 hr. mDCs were then washed and co-cultured with autologous CD4 T cells at a 1∶30 DC∶T cell ratio. After 1 hr brefeldin A was added and the cells were further incubated overnight. Cells were harvested and stained with antibodies to detect cytokine-producing CD4 T cells and analyzed by flow cytometry. Dot plots show live CD4 T cells and numbers indicate frequency of IFNα, TNFα, IL-2+ CD4 T cells. One representative experiment of four is shown.

Uninfected and HI IAV exposed mDCs had similar capacities to cross-present CMV to antigen-specific CD8 T memory cells. In contrast, IAV infected mDCs consistently produced several-fold lower frequency of proliferating CMV pp65-specific CD8 T cells (Figure 5A). The impaired ability of IAV infected mDCs to cross-present inactivated CMV to CD8 T cells was apparent over a range of CMV concentrations (Figure 5B) and DC∶T cell ratios (Figure 5C). Analysis of the CMV pentamer-negative, CFSE^low^ population in the absence of exogenous CMV also showed that the overall T cell response was more pronounced to HI IAV (48.6%) than to infectious IAV (15.9%) (Figure 5A, left panel). Furthermore, IAV infected and HI IAV stimulated mDCs loaded with pre-processed CMV peptide were comparable or superior to uninfected mDCs in their ability to expand CMV-specific CD8 T cells, consistent with a defect in antigen-processing capacity rather than in antigen-presentation (Figure 5D). Similar results were observed for cross-presentation of HI EBV or EBV infected cell extract (Figure S7). This suggests that IAV infected mDCs have an impaired capacity to cross-present both different sources of antigen (CMV and EBV) as well as different forms of antigen (inactivated virus and virus infected cells) to CD8 T cells as compared to uninfected mDCs.

We also compared the ability of uninfected, IAV infected, and HI IAV stimulated mDCs to present CMV to autologous CD4 T cells. Unlike cross-presentation to CD8 T cells, IAV infected and HI IAV exposed mDCs stimulated CMV-specific CD4 responses similarly (Figure 5E). This again indicates that IAV infected mDCs can function as APCs in general but that IAV infection selectively impairs the ability to cross-present antigen on MHCI to CD8 T cells.

### Neither differential cellular viability nor antigen load explain the decreased ability of IAV infected mDCs to cross-present

**Figure 6:**
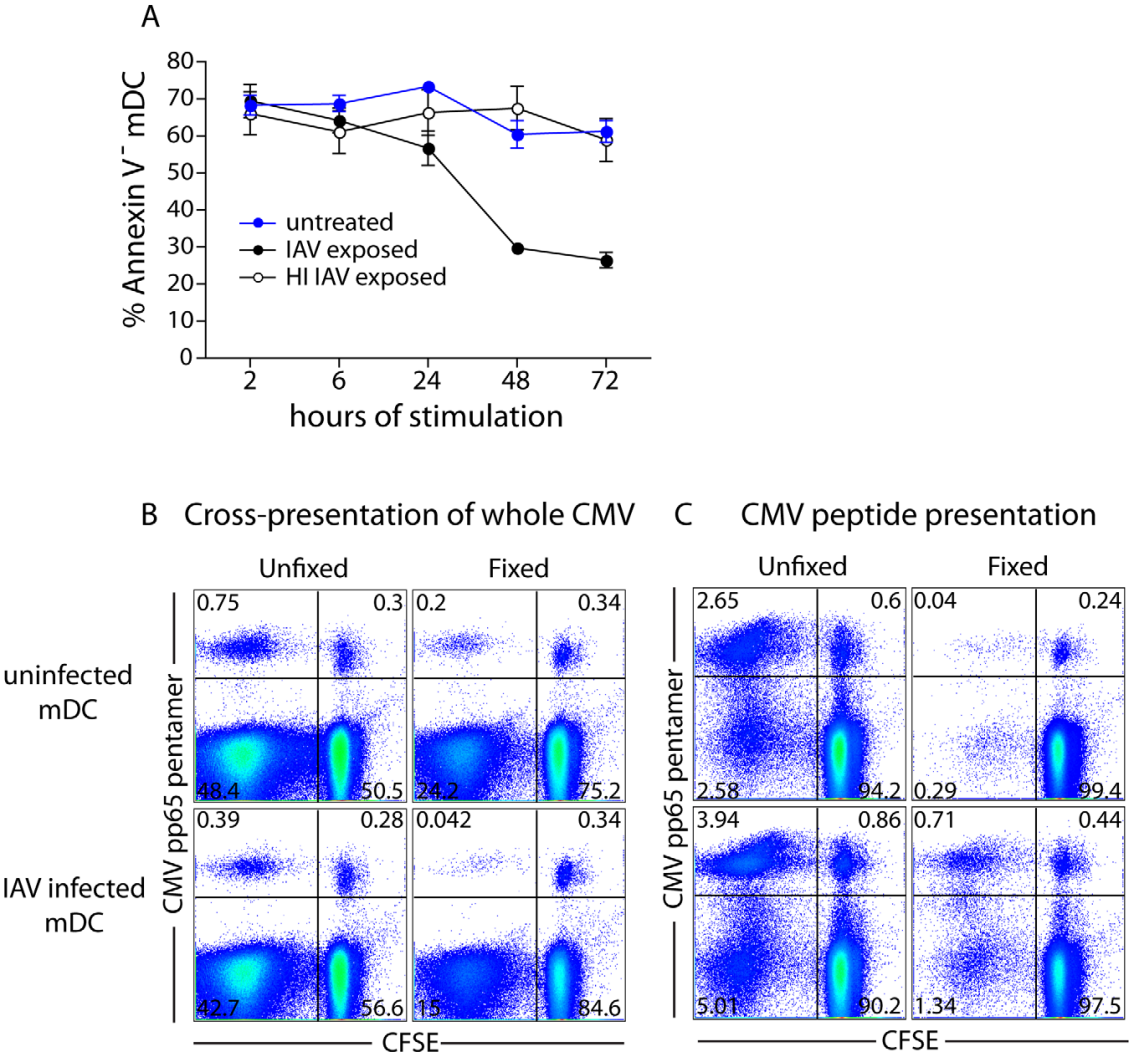
IAV-induced cell death does not explain difference in ability between IAV infected and uninfected mDCs to cross-present antigen to CD8 T cells. (**A**) mDCs were exposed to infectious IAV, HI IAV or left untreated. mDCs were collected at different time points and stained with Annexin V to assess cell viability. Graph shows average frequency ± SD of Annexin V negative (live) mDCs treated with nothing (blue), infectious IAV (black) or HI IAV (white) (n = 3). (**B**) mDCs were infected or not with infectious IAV for 2 hr, washed and exposed to 700 µg/mL whole, γ-irradiated CMV for an additional 6 hr. mDCs were then washed, split in two, and half of the DCs were fixed with 0.5% PFA for 10 min and washed extensively to remove any residual PFA before co-culture with autologous CFSE-labeled CD8 T cells at a 1∶30 DC∶T cell ratio for 10 days. Cells were harvested and stained with an HLA-A2 CMV pp65 pentamer and analyzed by flow cytometry. Dot plots show live CD8 T cells and numbers indicate frequency of positive CD8 T cells. One representative experiment of two is shown. (**C**) Same experiment as described in (B) but uninfected or IAV infected mDCs were loaded with 25 ng/mL pre-processed CMV peptide before fixation (or not) and co-cultured with autologous CD8 T cells at a 1∶30 DC∶T cell ratio. Dot plots show live CD8 T cells and numbers indicate frequency of positive CD8 T cells. One representative experiment of two is shown.

One explanation for the decreased ability of IAV infected mDCs to cross-present could be IAV-induced DC death. This explanation appeared unlikely since presentation of pre-processed peptide was similar between uninfected and IAV infected mDCs (Figure 5D), and IAV infected mDCs could activate CD4 T cells (Figures 4A and 5A). Assessing the viability of the mDCs in the co-cultures after 10 days, the time at which we measure T cell activation, is a challenge since DCs are in great minority and the majority of T cells that have not seen their cognate antigen have died. To investigate the role of IAV mediated cell death, mDCs were exposed to infectious IAV or HI IAV and the frequency of dead mDCs was compared to untreated mDCs by Annexin V staining. The viability of all mDCs was comparable at 2 hr and 6 hr after virus exposure (Figure 6A). At later time points (days), the viability of IAV infected mDCs was reduced compared to untreated or HI IAV exposed mDCs (Figure 6A), in line with the cytopathic effects of IAV and similar to what has been described by others [36]–[37]. However, as most of the antigen processing and presentation to CD8 T cells probably occurs within the first 24 hr of antigen capture [43], these data suggest that IAV induced cell death alone is not a likely explanation for the difference in cross-presentation observed.

Another way to address the role of DC viability in the observed difference in cross-presentation is to stop antigen processing after 8 hr by fixing the DCs with paraformaldehyde (PFA) and then analyze CD8 T cell expansion. Since fixed cells do not present antigen as effectively as viable cells, the overall frequency of expanded CMV pp65-specific CD8 T cells was lower than in the cultures with live DCs (Figure 6B). Nevertheless, the relative difference remained the same: fixed IAV infected mDCs were poorer at expanding CD8 T cells than fixed uninfected mDCs. In contrast, fixed IAV infected mDCs loaded with pre-processed peptide were better than fixed uninfected mDCs at expanding CD8 T cells (Figure 6C). This is in agreement with IAV induced mDC maturation and MHCI upregulation (Figure 2C).

**Figure 7:**
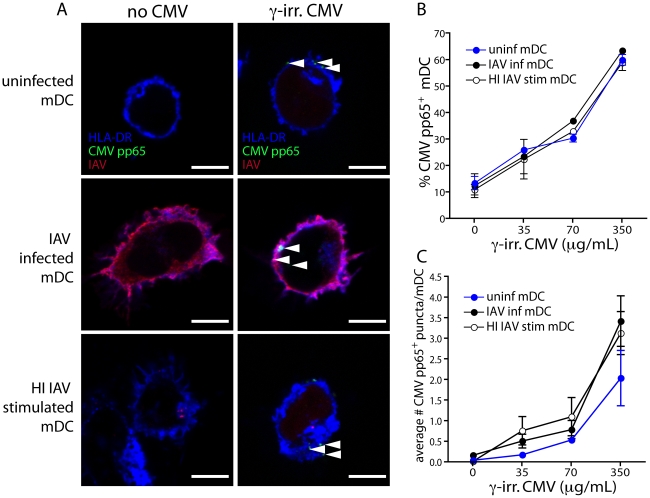
IAV infected and uninfected mDCs have similar CMV antigen load. (**A**) mDCs were exposed to infectious IAV, HI IAV or untreated for 4 hr, washed and exposed to increasing doses of whole, γ-irradiated CMV for an additional 3 hr. DCs were then washed, adhered to coverslips and fixed with PFA. After permeabilization, samples were stained for CMV pp65 (green), IAV (red) and HLA-DR (blue). The entire volume of each cell was analyzed using confocal microscopy (100× 1.47NA oil objective, 7× digital zoom) and one single optical slice is shown through the center of the cell with arrowheads pointing to CMV pp65+ structures. Scale bar 5 µm. (**B**) The frequency of CMV pp65+ uninfected mDCs (blue), IAV infected mDCs (black) or HI IAV stimulated mDCs (white) after 3 hr of CMV exposure was determined by analyzing entire z-stacks of DCs and counting the number of cells that had virus associated with them. The graph shows average frequency of CMV positive DCs ± SD, with 100 cells analyzed per donor and condition (n = 3). (**C**) The average number of CMV pp65+ puncta per cell after 3 hr of CMV exposure was determined by analyzing entire z-stacks of DCs and counting the number of green (CMV) puncta per nucleated cell, assessed by DAPI staining. The graph shows average frequency of CMV positive DCs ± SD, with 100 cells analyzed per donor and condition (n = 3).

Another explanation for the reduced ability of IAV infected mDCs to expand CMV-specific CD8 T cells could be poorer uptake of CMV particles. To address this possibility, we exposed uninfected, IAV infected and HI IAV stimulated mDCs to inactivated CMV particles and quantified the frequency of CMV-containing DCs by immunofluorescence (Figure 7A). The frequency of mDCs containing CMV particles was very similar over a range of CMV doses, irrespective of IAV infectivity (Figure 7B). We counted the number of CMV pp65-positive puncta per mDC in each condition. Again, uninfected, IAV infected and HI IAV stimulated mDCs were comparable, although there was a slight tendency for IAV infected mDCs to exhibit a higher average number of CMV pp65+ particles per cell (Figure 7C). These results suggested that there was no difference in antigen load between uninfected and IAV infected mDCs. Taken together, we conclude the reduced ability of IAV infected mDCs to cross-present likely depends on interference downstream of antigen uptake induced by viral replication and independent of DC maturation.

## Discussion

We have investigated the susceptibility of human primary DC subsets to IAV infection, including the functional consequences of IAV infection on their ability to process and present antigens to activate T cells. Although both mDCs and pDCs could internalize IAV, only mDCs supported IAV protein synthesis. Yet, IAV infected mDCs were less efficient at stimulating CD8 T cell responses in vitro when compared to uninfected mDCs. Indeed, cross-presentation of exogenous IAV M1 derived from internalized HI IAV was ∼300-fold more efficient when normalized to the total amount of mDC-associated antigen in infected vs. uninfected DCs. In addition, IAV infected mDCs also had a reduced capacity to present a second exogenous antigen, either inactivated CMV or EBV virions or infected cells, to CD8 T cells as compared to uninfected mDCs. Furthermore, we found that mDCs were more efficient at activating and expanding IAV-specific CD8 T cells than pDCs after exposure to either infectious or HI IAV.

Although previous studies have demonstrated that human DCs present infectious and inactivated IAV to CD4 and CD8 T cells [32]–[33], [36], [44], the relative efficiency of these two processes and the functional implication of any difference have not been studied in detail. In mouse models, the importance of CD8α+ DCs and cross-presentation in control of virus infection is well-established [15]. Furthermore, different virus infections in mouse including vaccinia [20], MCMV [22] and HSV-1 [21] have been reported to rely on cross-presentation rather than direct presentation for CD8 T cell responses and clearance of infection, although this conclusion has recently been challenged [19]. Our observation that cross-presentation can be more efficient than direct presentation on MHCI to CD8 T cells also in a human system extends earlier findings and underscores their relevance. The fusogenic capacity of IAV is required for optimal CD8 T cell activation [32], and may be crucial for efficient cross-presentation, since it likely removes the rate limiting step of endosomal egress. Our work further shows that IAV infected mDCs are impaired in their ability to cross-present a second antigen to CD8 T cells when compared to uninfected mDCs or mDCs exposed to replication-incompetent HI IAV. Importantly, it seems that replicating IAV, rather than the mere presence of IAV and subsequent DC maturation, affects the ability of mDCs to process and present antigen on MHCI for activation and expansion of CD8 T cells. To pinpoint which viral protein(s) and what specific cellular target(s) is affected will be key in future studies.

We have also demonstrated that in physiologically relevant DCs, cross-presentation can provide a more effective strategy for CD8 T cell stimulation than presentation of endogenous antigen by infected DCs, similar to what has been observed for other viruses in mouse experimental models [20]–[22]. Thus, not only is there no need for DCs to be infected by the viruses whose antigens they present, but also infection may actually suppress the initiation of adaptive responses. In future studies, it will be important to verify the relevance of our data using human lung DCs and/or in clinical studies that take into account the complex interaction of cells during IAV infection in vivo.

We found that pDCs were resistant to IAV infection despite significant virus internalization, confirming and extending previous reports [33]–[34], [36]–[37], [45]–[46]. pDCs responded to IAV exposure by secreting large amounts of IFNα, but showed only modest upregulation of co-stimulatory molecules compared to TLR7/8L stimulation. In our hands, pDCs were less potent than mDCs at inducing CD8 T cell activation after acquiring a large antigen that requires processing into peptides before loading onto MHCI. This was probably due to a lower expression of co-stimulatory molecules and therefore weaker DC-T cell interaction and/or a reduced capacity to process large antigens compared to mDCs, rather than a lack of viral antigen available for presentation, as pDCs carrying abundant IAV NP are unable to activate IAV-specific T cells [30]. Previous studies have reported that human pDCs are similar [36] or superior [35] in their ability to present antigen to CD8 T cells as compared to human mDCs. The lack of clear consensus may partly be explained by differences in maturation/phenotype of the pDCs as well as length of exposure, and dose of IAV, as it has recently been reported that the timing of pDC stimulation and route of antigen uptake affect the ability of pDCs to present antigens [47].

It is well documented that IAV infection renders infected individuals more prone to secondary bacterial infections, but the importance of CD8 T cell response to control and clear extracellular bacterial infections is unclear. IAV infection has an immunomodulatory effect that is thought to promote an increased susceptibility to secondary infections [24], [26]. The impact of IAV induced immunomodulation combined with an impaired ability to cross-present subsequently encountered antigens might act together to compromise a proper immune response to secondary pathogens. Systemic injection of TLR ligands results in reduced cross-presentation of a subsequently encountered antigen [18]. While this was suggested to be a consequence of systemic DC maturation, we recently showed that reduced antigen-presentation in vivo after systemic TLR injection could also be a consequence of the antigen not reaching DCs in the spleen due to alterations in splenic blood flow [48]. Previous studies using monocyte-derived DCs have shown that IAV infection induces suboptimal maturation of the cells with respect to upregulation of co-stimulatory molecules and secretion of cytokines as compared to LPS stimulation [49]. Using recombinant IAV that did not encode the multifunctional viral protein NS1, the authors found that NS1 has an inhibitory effect on expression of several genes involved in monocyte-derived DC maturation and migration, including the pro-inflammatory cytokines IL-6 and TNFα [49]. In our hands, primary mDCs do not show a defect in their ability to upregulate co-stimulatory molecules in response to IAV as compared to TLR stimulation, but did indeed show lower secretion of pro-inflammatory cytokines. In any event, altered DC maturation seems unlikely to fully explain the defect in mDC cross presentation of a second antigen, since mDCs stimulated with HI IAV, which mature to the same extent as IAV infected mDCs, were found to retain their ability to cross-present. In future studies, it will be important to use recombinant IAV strains in which different viral proteins have been mutated or deleted to study their potential impact on DC maturation and antigen-presentation on the protein level, as well as in functional assays as outlined in the present study.

Finally, our findings shed light on how uninfected human DCs, rather than IAV infected DCs, may be crucial for processing and presentation of IAV antigen to initiate anti-viral immunity. Even if infected DCs can present antigen, cross-presentation facilitated by uninfected DCs may be sufficient or even required for induction of anti-viral immune responses. While the in vivo situation is much more complicated, the in vitro results presented here do create the conceptual possibility that the same situation applies in vivo. Indeed, it was unexpected that uninfected mDCs cross-present viral antigens more efficiently than IAV infected DCs present endogenously synthesized antigens. Thus, IAV infection not only inhibits cross-presentation of subsequently encountered antigens, but also acts to diminish direct presentation. As a result, it is now of interest to determine the mechanism of both forms of inhibition.

Besides DC death, other potential contributors may include the partial reduction in host cell protein synthesis following IAV infection or a direct inactivation of the antigen processing machinery, as observed for medium to large DNA viruses that cause chronic infections [50]–[51]. As discussed above, the multifunctional IAV protein NS1 is an important virulence factor associated with the suppression of innate immunity [52]–[54]. The major function of NS1 is to antagonize the type I IFN mediated host response. Current evidence suggests that NS1 can limit IFNβ production both on the pre- and post-transcriptional level. While most IAV strains can utilize both strategies, some strains may have lost one of these mechanisms naturally or as a consequence of passage in the laboratory [53]. NS1 not only prevents the activation of IRF3, a transcription factor involved in IFNβ induction (pre-transcriptional), but can also block the expression of cellular genes such as MxA at the post-transcriptional level, and thereby IFN gene expression. In contrast to more recent human strains of IAV like A/TX/91 (TX), NS1 expressed by A/PR/8 (PR8), a widely used laboratory IAV strain, can only limit pre-transcriptional events of IFNβ induction [54]. Monocyte-derived DCs infected with IAV/TX displayed higher viral replication but reduced capacity to induce IFNγ secretion in allogeneic naive CD4 T cells compared to monocyte-derived DCs infected with IAV/PR8 [55]. Monocyte-derived DCs infected with NS1 deleted versions of the two virus strains were comparable in their ability to induce IFNγ secretion in allogeneic naive CD4 T cells [55], suggesting that a more recent human isolate of IAV (TX) is a more potent inhibitor of DC function than a laboratory adapted strain (PR8). In addition, recent data using human lung epithelial cells indicate that NS1 specifically suppresses the expression of several genes involved in IFN-stimulated MHCI antigen presentation and immune-proteasome activation during IAV infection [56]. Another potential viral protein to consider in this context is the most recently discovered IAV protein, PB1-F2 [57]. PB1-F2 is a virulence factor described to contribute to pathogenesis of IAV as well as secondary bacterial infections [58]–[60]. Taken together, these studies have contributed significantly to our initial understanding of how individual IAV proteins may impact the immune response to IAV and they also highlight the importance of studying a wider selection of IAV strains. Whether NS1 and/or PB1-F2 also affect the ability of IAV infected primary DCs to cross-present is a relevant question that merits further investigation. A deeper understanding of how IAV infection of human DCs impairs their function may prove to be useful for improved vaccine design or therapeutic approaches to enhance endogenous responses.

## Materials and Methods

### Ethics statement

This study was approved by the Genentech Institutional Review Board. Written informed consent was obtained from all human participants.

### Isolation and culture of cells

Our procedures for isolation of subsets of DCs and T cells from blood have been described previously [61]. Briefly, healthy blood donors underwent automated leukapheresis and enriched populations of lymphocytes and monocytes were obtained by counterflow centrifugal elutriation. DCs were isolated from elutriated monocytes using magnetic bead isolation followed by sequential separation on AutoMacs (Miltenyi Biotec). The BDCA-4 and the CD1c isolation kits were used for isolation of pDCs and mDCs, respectively. pDCs and mDCs were cultured at 1×10^6^ cells/ml in complete medium (RPMI 1640 Glutamax supplemented with 1% streptomycin and penicillin, 1% HEPES (all Invitrogen), 10% fetal bovine serum (Gibco)) in the presence of recombinant human IL-3 (10 ng/ml, R&D Systems) or GM-CSF (2 ng/ml, PeproTech). T cells were isolated from elutriated lymphocytes by negative selection and separation on AutoMacs. T cells were cultured at 10×10^6^ cells/ml in complete medium and rested overnight before use.

### IAV strains

Influenza A/NWS/33 and Influenza A/PR/8/34 strains (ATCC) were propagated in MDCK cells. Supernatants were concentrated by ultracentrifugation and resuspended in RPMI. Influenza A/X31 was propagated in chicken eggs, purified and concentrated on sucrose gradients (Virapur). Mock infected supernatants and allantoic fluid were processed in the same manner and used as controls to exclude any non-specific activation of DCs (data not shown). TCID50 for all IAV strains was determined by infecting a light monolayer of MDCKs in the presence of trypsin and monitoring the cytopathic effect. DCs were infected with 600,000 infectious particles (assessed in MDCK plaque assay) of IAV per 1,000,000 DCs (0.6 MOI). This dose of IAV resulted in 50–95% IAV+ mDCs after 24 hr of exposure. Virus was replication incompetent after heat-inactivation at 56°C for 30 min. Unless otherwise stated in the text, IAV refers to IAV/X31.

### IAV infection and stimulation of DCs

DCs were exposed to IAV, washed twice in RPMI and infection was monitored using an anti-IAV rabbit polyclonal (Pinda, Dr. Ari Helenius, ETH Zurich, Switzerland) or anti-nucleoprotein (NP) antibody (clone A3, Chemicon) and flow cytometry (FACSCanto II, BD Biosciences). Alternatively, infected DCs were allowed to adhere to alcian blue (Sigma) coated glass coverslips for 20 min at 37°C, fixed with 4% paraformaldehyde (PFA) (Electron microscopy sciences) for 20 min at room temperature and permeabilized with 0.05% saponin (Sigma), stained with antibodies and analyzed by immunofluorescence confocal microscopy (Leica TCS SP5, Leica Microsystems). To prevent IAV infection, 20 mM NH_4_Cl was added before IAV.

### DC phenotype and cytokine secretion

After IAV infection, DCs were harvested, washed twice and surface stained with antibodies against (CD14 (MφP9), CD11c (B-ly6), CD123 (9F5), CD86 (FUN-1), CD40 (5C3), HLA-ABC (W6/32) all BD Biosciences) or HLA-DR (L243, Biolegend). DCs were washed, fixed and analyzed by flow cytometry. Supernatants were harvested and cytokines were measured by ELISA (IFNα; PBL Interferon Source) or Luminex (Biorad). MxA expression was determined using a mouse anti-MxA monoclonal antibody (clone M143, Dr. Otto Haller, University of Freiburg, Germany) and flow cytometry or immunofluorescence confocal microscopy.

### Presentation of IAV to memory CD4 T by DCs

After 4 hr of IAV exposure, DCs were washed and co-cultured with autologous CD4 T cells at different DC∶T cell ratios. After 1 hr, GolgiPlug containing Brefeldin A (BD Biosciences) was added and the cells were further incubated overnight. Cells were harvested and stained with surface antibodies against CD4 (SK3), CD3 (SK7), CD8 (SK1), CD14 (all BD Biosciences) and HLA-DR, followed by fixation and permeabilization for 10 min using BD cytofix/cytoperm (BD Biosciences). Cells were stained intracellularly with antibodies against IFNγ (B27, BD), TNFα (MAb11, BD) and IL-2 (MQ1-17H12, Caltag laboratories) and analyzed by flow cytometry.

### Presentation of IAV to memory CD8 T by DCs

DCs isolated from HLA-A2+ donors were exposed to IAV or loaded with 0.25–250 ng/mL pre-processed peptide for 4 hr, washed and co-cultured with autologous CD8 T cells labeled with 0.25 µM CFSE (Molecular Probes). As a positive control, the TCR superantigen Staphylococcal enterotoxin B (1 µg/ml, Sigma) was used. HLA-A2 restricted HIV-1 gag pre-processed peptide (SLYNTVATL) was used as an irrelevant pre-processed peptide control (ProImmune). After 10 days, cells were harvested and stained with HLA-A2 Influenza M1 (GILGFVFTL) pentamer (ProImmune) for 15 min at room temperature followed by labeling with antibodies against CD3, CD8, CD14, CD19 (SJ25C1), CD11c (B-ly6) (BD Biosciences), fixation and analysis by flow cytometry.

### Presentation of CMV or EBV to memory CD8 T by uninfected or IAV infected mDCs

HLA-A2+ mDCs were exposed to IAV for 4 hr, washed and loaded with 7–700 µg/mL of total protein whole, inactivated CMV (Microbix) or 0.25–250 ng/mL pre-processed HLA-A2 CMV pp65 peptide (NLVPMVATV) for an additional 3 hr. DCs were washed and co-cultured with autologous CD8 T cells labeled with CFSE. After 10 days, cells were harvested and stained with HLA-A2 CMV pp65 (NLVPMVATV) pentamer (ProImmune) followed by labeling with antibodies against CD3, CD8, CD19, CD11c, CD14, fixation and analysis by flow cytometry. Alternatively, mDCs were loaded with 200 µg/mL total protein from whole, heat-inactivated EBV (Virusys) or 200 µg/mL total protein cell extract from EBV infected or control cells (Virusys) or 0.25–250 ng/mL pre-processed HLA-A2 EBV BMLF-1 peptide (GLCTLVAML) for 3 hr, washed and co-cultured with CD8 T cells. After 10 days, cells were harvested and stained with HLA-A2 EBV BMLF-1 (GLCTLVAML) pentamer (ProImmune) and surface antibodies as described above. The CMV and EBV antigen preparations were titrated to find a dose that was not toxic to the cells yet adequate to activate memory T cells.

### Presentation of CMV to memory CD4 T by uninfected or IAV infected mDCs

After 4 hr IAV exposure, mDCs were washed and pulsed with 7–700 µg/mL of total protein from whole, inactivated CMV or overlapping pre-processed peptides to CMV pp65, 15-mers overlapping by 11 (2.5 µg of peptide per mL, ProImmune) for 3 hr. DCs were washed and co-cultured with autologous CD4 T cells at a 1∶30 DC∶T cell ratio. After 2 hr, GolgiPlug was added and the cells were incubated overnight. Cells were harvested and stained with surface antibodies against CD4, CD3, HLA-DR, CD14, CD8, followed by fixation and permeabilization. Cells were subsequently stained with antibodies against IFNγ, TNFα and IL-2, and analyzed by flow cytometry.

### IAV antigen load in mDCs

After 8 hr of IAV exposure, mDCs were harvested and lysed in SDS lysis buffer (1% SDS, 20 mM Tris pH 7.5 and protease inhibitors (Roche)). DNA was shed mechanically and lysates were snap frozen on dry ice. Lysates were run on a 4–12% Bis-Tris reducing gel, transferred to a PVDF membrane and blotted for viral proteins with the anti-IAV polyclonal Pinda. GAPDH was used as loading control.

### mDC viability after IAV infection

mDCs were exposed to infectious IAV or HI IAV or left untreated. DCs were harvested, washed twice in ice-cold PBS, resuspended in 1× binding buffer and stained with Annexin V and propidium iodide (BD Biosciences) and analyzed by flow cytometry within one hour of processing.

### CMV antigen load in uninfected and IAV infected mDCs

After 4 hr of IAV exposure, DCs were washed and pulsed with 7–700 µg/mL of total protein whole, inactivated CMV (Microbix) for 3 hr. DCs were washed twice in complete medium, adhered to coverslips, fixed and permeabilized. DCs were stained with antibodies against IAV (Pinda), CMV pp65 (clones 2+6, Leica) and HLA-DR and mounted with Prolong Gold containing DAPI (Molecular Probes). Samples were analyzed by immunofluorescence confocal microscopy.

### Statistical analyses

Statistical significance was assessed using paired *t* test and considered significant at *P* value less than 0.05.

## Supporting Information

Figure S1 **Neither mDCs nor pDCs support production of infectious IAV.** (**A**) DCs were exposed to IAV in the absence or presence of NH_4_Cl for 1 hr, washed 3 times to remove any free virus and cultured for 24 hr with or without NH_4_Cl. Supernatants were collected and TCID50 was determined by infecting a light monolayer of MDCKs in the presence of trypsin and monitoring the cytopathic effect. For comparison, the input IAV was included in the assay. Graph shows mean±SD (n = 3). (**B**) Susceptibility of mDCs and pDCs to different IAV strains. pDCs (red) and mDCs (blue) were exposed to IAV/X31, IAV/PR8 or IAV/WS in the absence or presence of NH_4_Cl for 24 hr. DCs were harvested and stained with an anti-nucleoprotein antibody to assess the frequency of IAV infected DCs by flow cytometry. Graph shows average frequency of NP+ DCs ± SD (n = 3).
(TIF)

Figure S2 **pDCs constitutively express high levels of the anti-viral type I interferon inducible protein MxA, while mDCs upregulate MxA expression upon maturation.** (**A**) Localization of MxA (green) in pDCs and mDCs after 24 hr of culture with or without stimulation with LPS or IFNα was analyzed by immunofluorescence and confocal microscopy. Images show DCs in bright field and nuclei are stained with DAPI (blue). 63× objective, 8× digital zoom. Scale bar 5 µm. (**B**) mDCs were stimulated with LPS or IFNα or left untreated overnight. The following day mDCs were exposed to IAV in the presence or absence of NH_4_Cl for 6 hr and the frequency of IAV+ mDCs was determined by intracellular staining and flow cytometry. Dot plots show live CD11c+ CD14− mDCs and numbers indicate frequency of positive mDCs. One representative donor of 3.
(TIF)

Figure S3 **IFNα secretion from mDCs and pDCs in response to different IAV strains.** pDCs (red) and mDCs (blue) were exposed to IAV/X31, IAV/PR8 or IAV/WS in the absence or presence of NH_4_Cl for 24 hr. Supernatants were harvested and analyzed by ELISA to assess the concentration of secreted IFNα. Graph shows average concentration of secreted IFNα ± SD (n = 3).
(TIF)

Figure S4 **Cytokine secretion from mDCs and pDCs in response to IAV.** pDCs (red) and mDCs (blue) were exposed to infectious IAV, HI IAV or TLR7/8L in the presence or absence of NH_4_Cl and the levels of secreted TNFα (**A**), IL-6 (**B**), MIP-1α (**C**), IL-1β (**D**), IL-12 p70 (**E**) and IL-10 (**F**) were determined by ELISA. The graphs show mean ± SD (n = 3).
(TIF)

Figure S5 **Number IAV structures per mDC.** mDCs were exposed to IAV for 1 hr, washed 3 times to remove free virus and allowed to adhere to coverslips. Cells were surface stained for HLA-DR, fixed and permeabilized and stained using an anti-IAV antibody. The entire volume of each cell was analyzed using confocal microscopy (100× 1.47NA oil objective, 6× digital zoom) and 3D reconstructed in Imaris before counting IAV+ puncta in individual cells. The graph shows individual cells as circles, from two independent experiments. The line indicates the average number of IAV+ structures per mDC in each experiment.
(TIF)

Figure S6 **mDCs are superior at activating IAV-specific CD8 T cells compare to pDCs.** mDCs (**A**) and pDCs (**B**) were exposed to increasing doses of infectious IAV, HI IAV or left untreated for 4 hr, washed to remove free virus and co-cultured with autologous CFSE labeled CD8 T cells at different DC∶T cell ratios. After 10 days of co-culture, cells were harvested and stained with an HLA-A2 Influenza A M1 (GILGFVFTL) pentamer to detect Influenza M1-specific CD8 T cells and analyzed by flow cytometry. T cell proliferation was detected by CFSE dilution. Bar graphs show one representative donor of two.
(TIF)

Figure S7 **IAV infected mDCs cross-present EBV less efficiently to CD8 T cells than uninfected mDCs.** mDCs were infected with infectious IAV or not for 4 hr, washed to remove non-cell associated virus and exposed to (**A–B**) HI EBV, (**C–D**) EBV infected or control cell extract, or (**E–F**) increasing doses of EBV MBLF-1 peptide (GLCTLVAML) for an additional 3 hr. mDCs were then washed and co-cultured with autologous CFSE-labeled CD8 T cells at (**A–D**) different or (**E–F**) 1∶30 DC∶T cell ratios. After 10 days of co-culture, cells were harvested and stained with an HLA-A2 EBV BMLF-1 pentamer to detect EBV BMLF-1-specific CD8 T cells and analyzed by flow cytometry. T cell proliferation was detected by CFSE dilution. (**A, C, E**) The graph shows frequency of EBV pentamer+ CFSE^low^ CD8 T cells after co-culture with uninfected mDCs (blue) or IAV infected mDCs (black). (**B–F**) Dot plots show live CD8 T cells and numbers indicate frequency of positive CD8 T cells at (**B**) 1∶100 or (**D**) 1∶30 DC∶T cell ratio or (**F**) co-cultured with mDCs loaded with 250 ng/mL EBV peptide. One representative experiment of 3 is shown.
(TIF)
