# Supplementary figures and images for: Influenza A Virus Infection of Human Primary Dendritic Cells Impairs Their Ability to Cross-Present Antigen to CD8 T Cells
Source: PLoS Pathog. 2012 Mar 8;8(3):e1002572. doi: 10.1371/journal.ppat.1002572 (PMC3297599; doi:10.1371/journal.ppat.1002572)

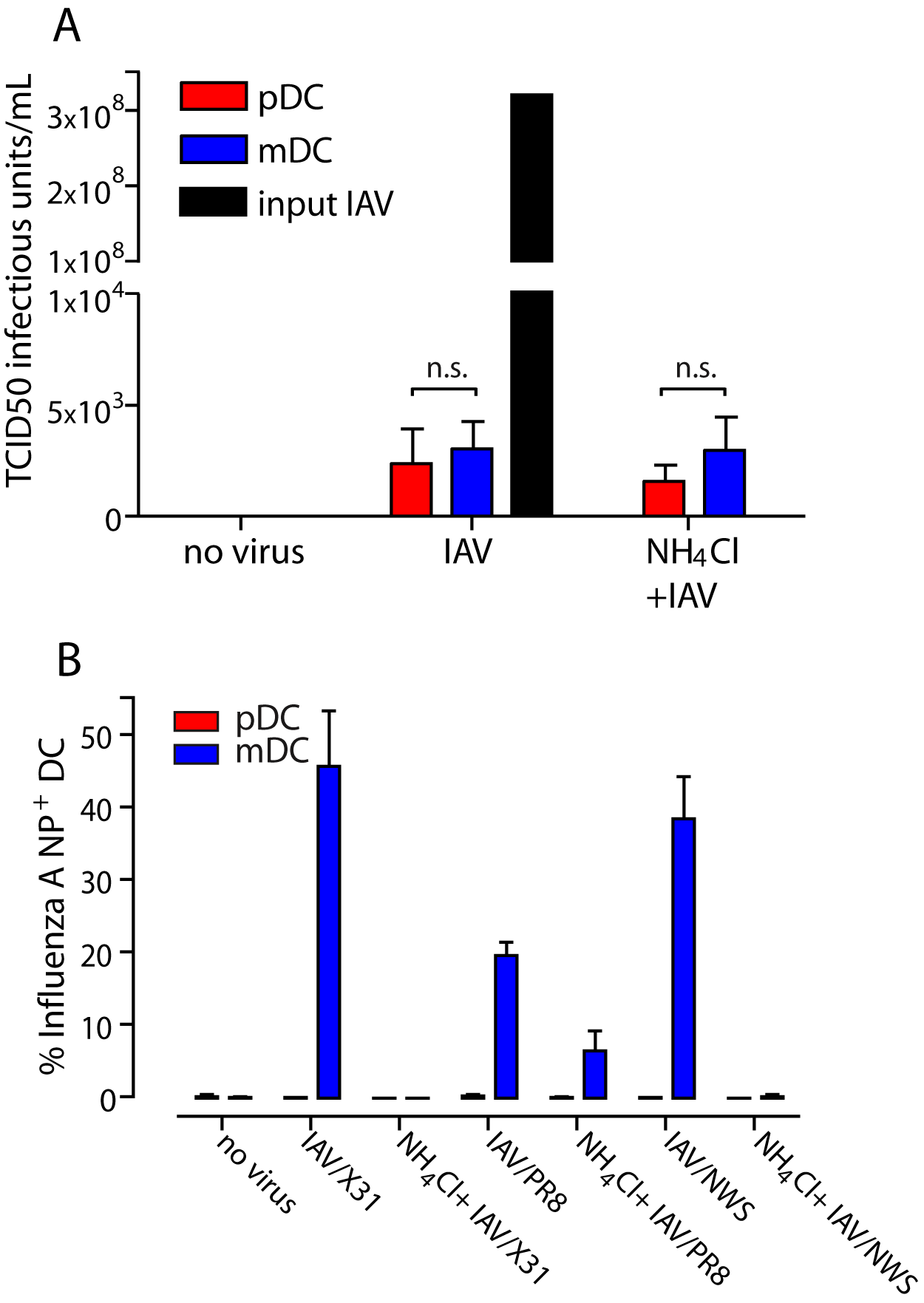

Supplement: Figure S1 — Neither mDCs nor pDCs support production of infectious IAV. (A) DCs were exposed to IAV in the absence or presence of NH4Cl for 1 hr, washed 3 times to remove any free virus and cultured for 24 hr with or without NH4Cl. Supernatants were collected and TCID50 was determined by infecting a light monolayer of MDCKs in the presence of trypsin and monitoring the cytopathic effect. For comparison, the input IAV was included in the assay. Graph shows mean±SD (n = 3). (B) Susceptibility of mDCs and pDCs to different IAV strains. pDCs (red) and mDCs (blue) were exposed to IAV/X31, IAV/PR8 or IAV/WS in the absence or presence of NH4Cl for 24 hr. DCs were harvested and stained with an anti-nucleoprotein antibody to assess the frequency of IAV infected DCs by flow cytometry. Graph shows average frequency of NP+ DCs ± SD (n = 3). (TIF) [file ppat.1002572.s001.tif]

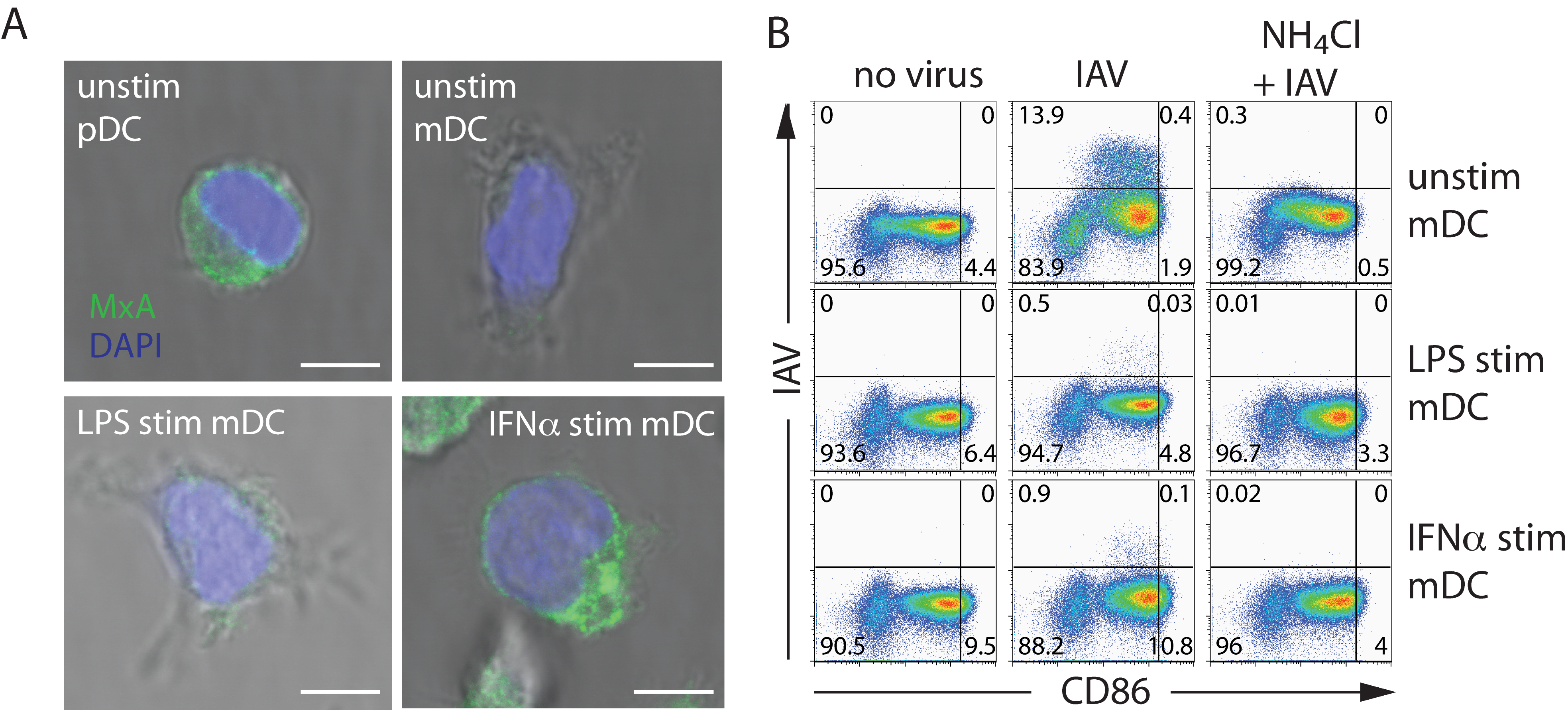

Supplement: Figure S2 — pDCs constitutively express high levels of the anti-viral type I interferon inducible protein MxA, while mDCs upregulate MxA expression upon maturation. (A) Localization of MxA (green) in pDCs and mDCs after 24 hr of culture with or without stimulation with LPS or IFNα was analyzed by immunofluorescence and confocal microscopy. Images show DCs in bright field and nuclei are stained with DAPI (blue). 63× objective, 8× digital zoom. Scale bar 5 µm. (B) mDCs were stimulated with LPS or IFNα or left untreated overnight. The following day mDCs were exposed to IAV in the presence or absence of NH4Cl for 6 hr and the frequency of IAV+ mDCs was determined by intracellular staining and flow cytometry. Dot plots show live CD11c+ CD14− mDCs and numbers indicate frequency of positive mDCs. One representative donor of 3. (TIF) [file ppat.1002572.s002.tif]

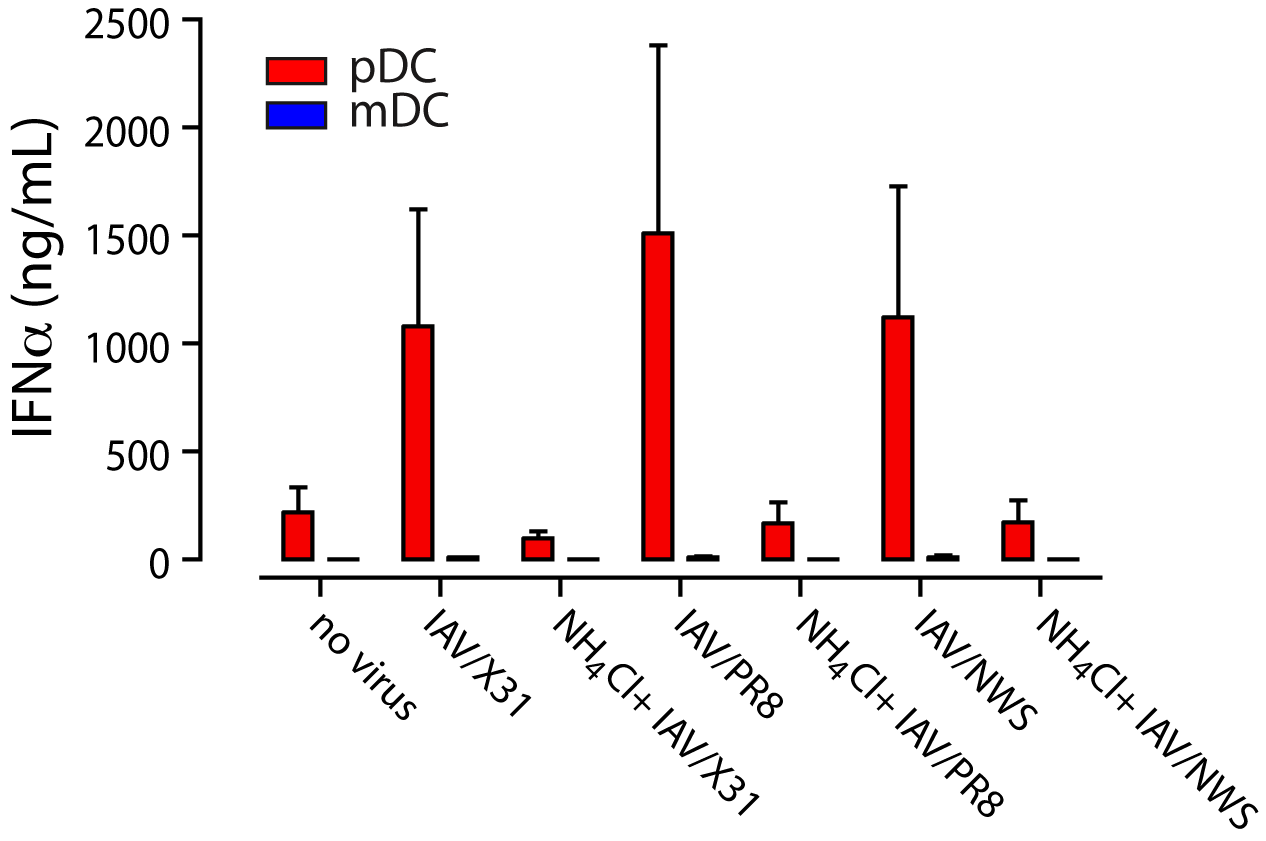

Supplement: Figure S3 — IFNα secretion from mDCs and pDCs in response to different IAV strains. pDCs (red) and mDCs (blue) were exposed to IAV/X31, IAV/PR8 or IAV/WS in the absence or presence of NH4Cl for 24 hr. Supernatants were harvested and analyzed by ELISA to assess the concentration of secreted IFNα. Graph shows average concentration of secreted IFNα ± SD (n = 3). (TIF) [file ppat.1002572.s003.tif]

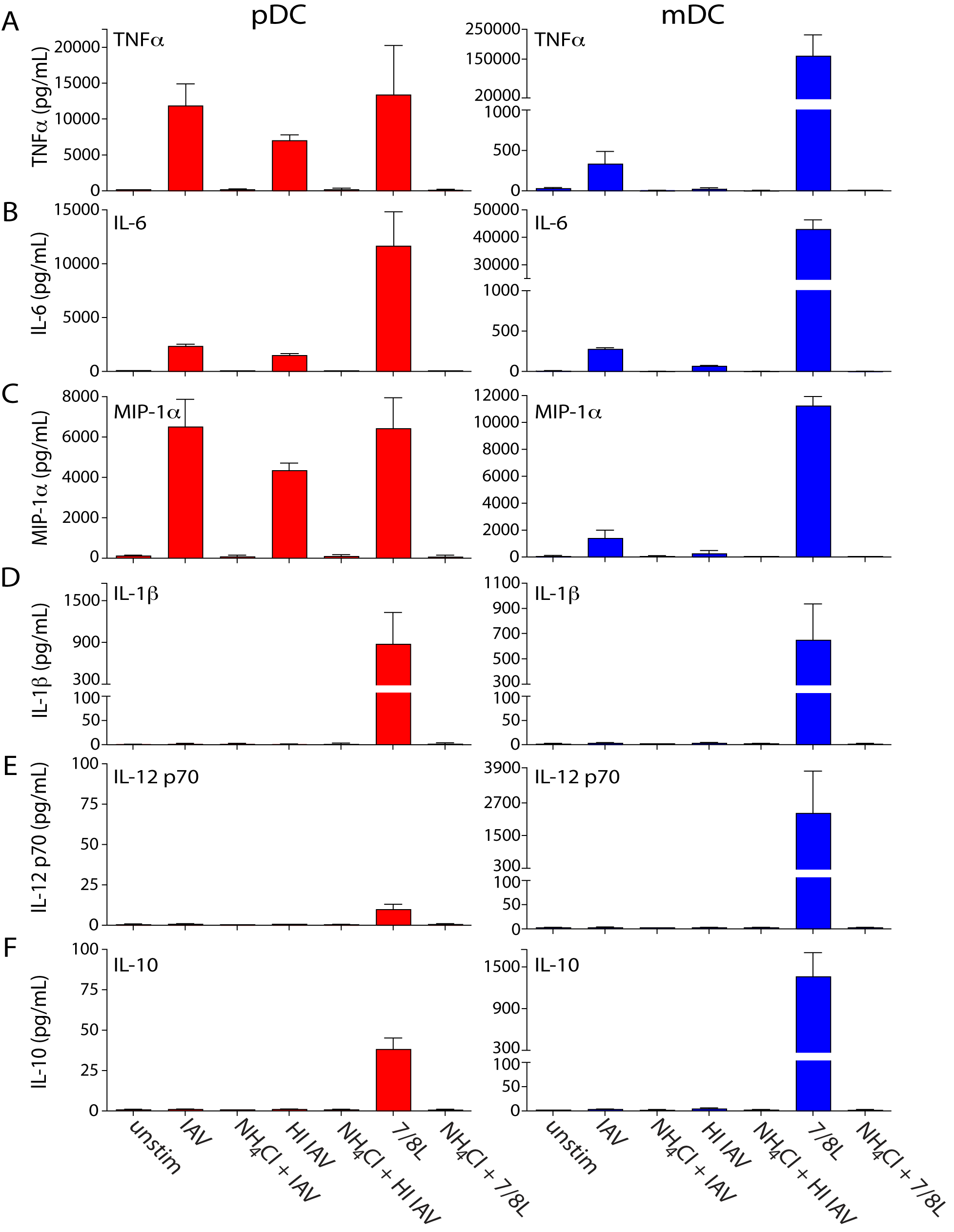

Supplement: Figure S4 — Cytokine secretion from mDCs and pDCs in response to IAV. pDCs (red) and mDCs (blue) were exposed to infectious IAV, HI IAV or TLR7/8L in the presence or absence of NH4Cl and the levels of secreted TNFα (A), IL-6 (B), MIP-1α (C), IL-1β (D), IL-12 p70 (E) and IL-10 (F) were determined by ELISA. The graphs show mean ± SD (n = 3). (TIF) [file ppat.1002572.s004.tif]

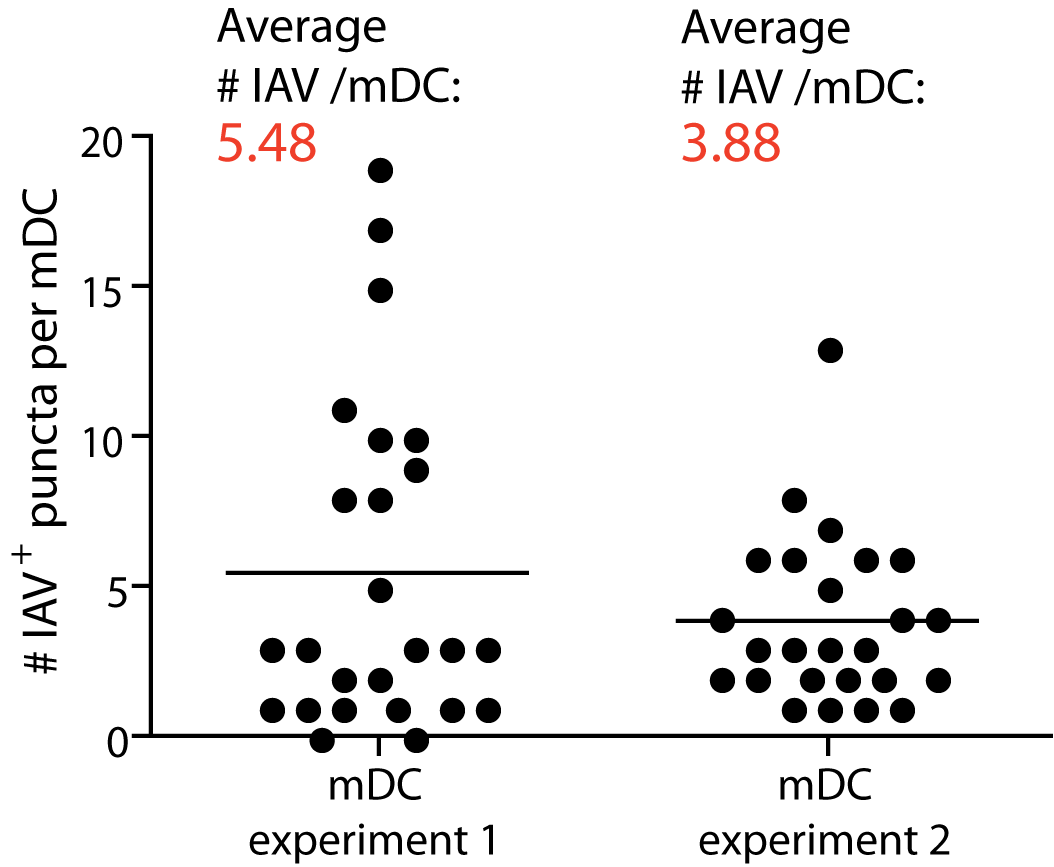

Supplement: Figure S5 — Number IAV structures per mDC. mDCs were exposed to IAV for 1 hr, washed 3 times to remove free virus and allowed to adhere to coverslips. Cells were surface stained for HLA-DR, fixed and permeabilized and stained using an anti-IAV antibody. The entire volume of each cell was analyzed using confocal microscopy (100× 1.47NA oil objective, 6× digital zoom) and 3D reconstructed in Imaris before counting IAV+ puncta in individual cells. The graph shows individual cells as circles, from two independent experiments. The line indicates the average number of IAV+ structures per mDC in each experiment. (TIF) [file ppat.1002572.s005.tif]

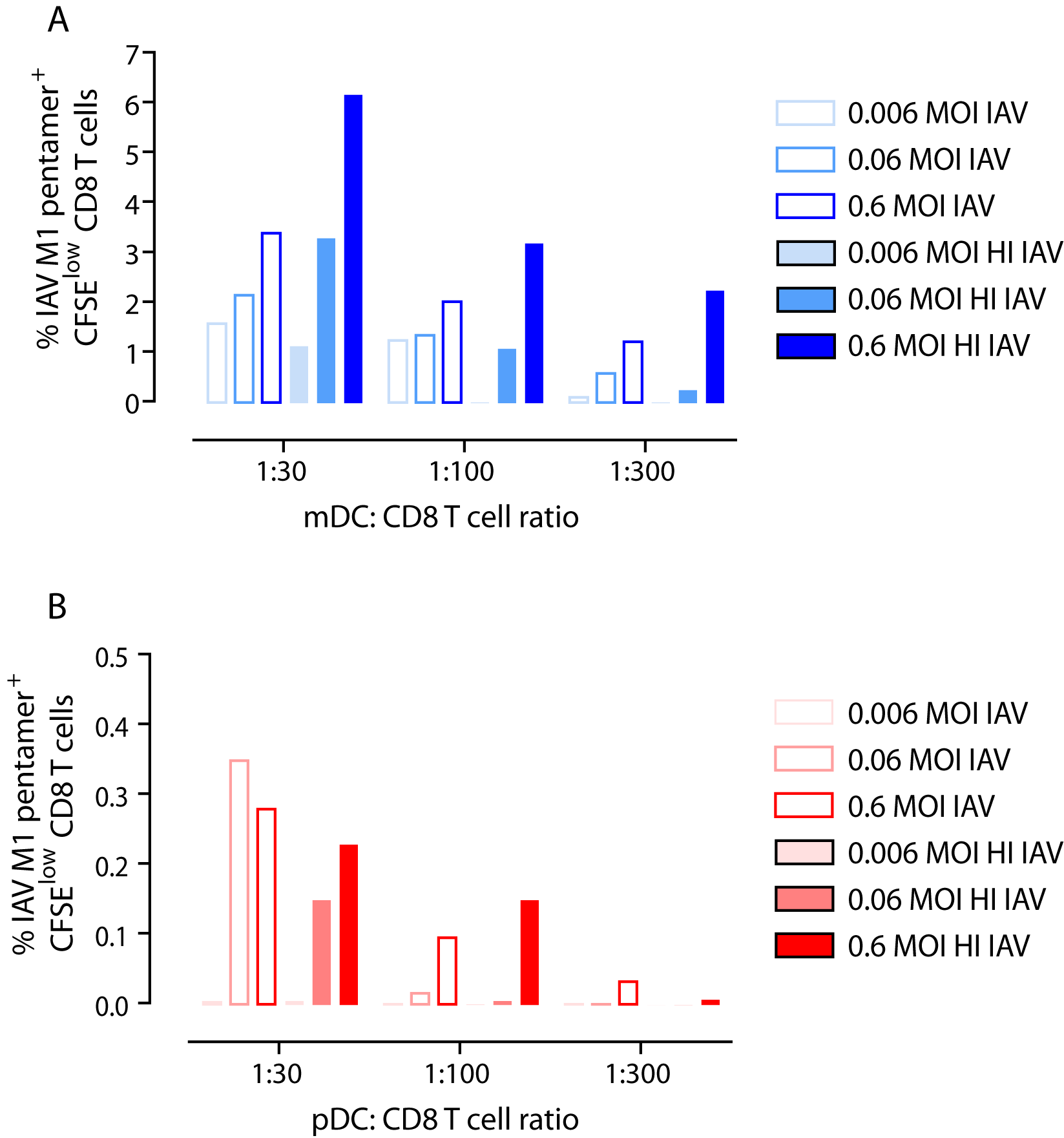

Supplement: Figure S6 — mDCs are superior at activating IAV-specific CD8 T cells compare to pDCs. mDCs (A) and pDCs (B) were exposed to increasing doses of infectious IAV, HI IAV or left untreated for 4 hr, washed to remove free virus and co-cultured with autologous CFSE labeled CD8 T cells at different DC∶T cell ratios. After 10 days of co-culture, cells were harvested and stained with an HLA-A2 Influenza A M1 (GILGFVFTL) pentamer to detect Influenza M1-specific CD8 T cells and analyzed by flow cytometry. T cell proliferation was detected by CFSE dilution. Bar graphs show one representative donor of two. (TIF) [file ppat.1002572.s006.tif]

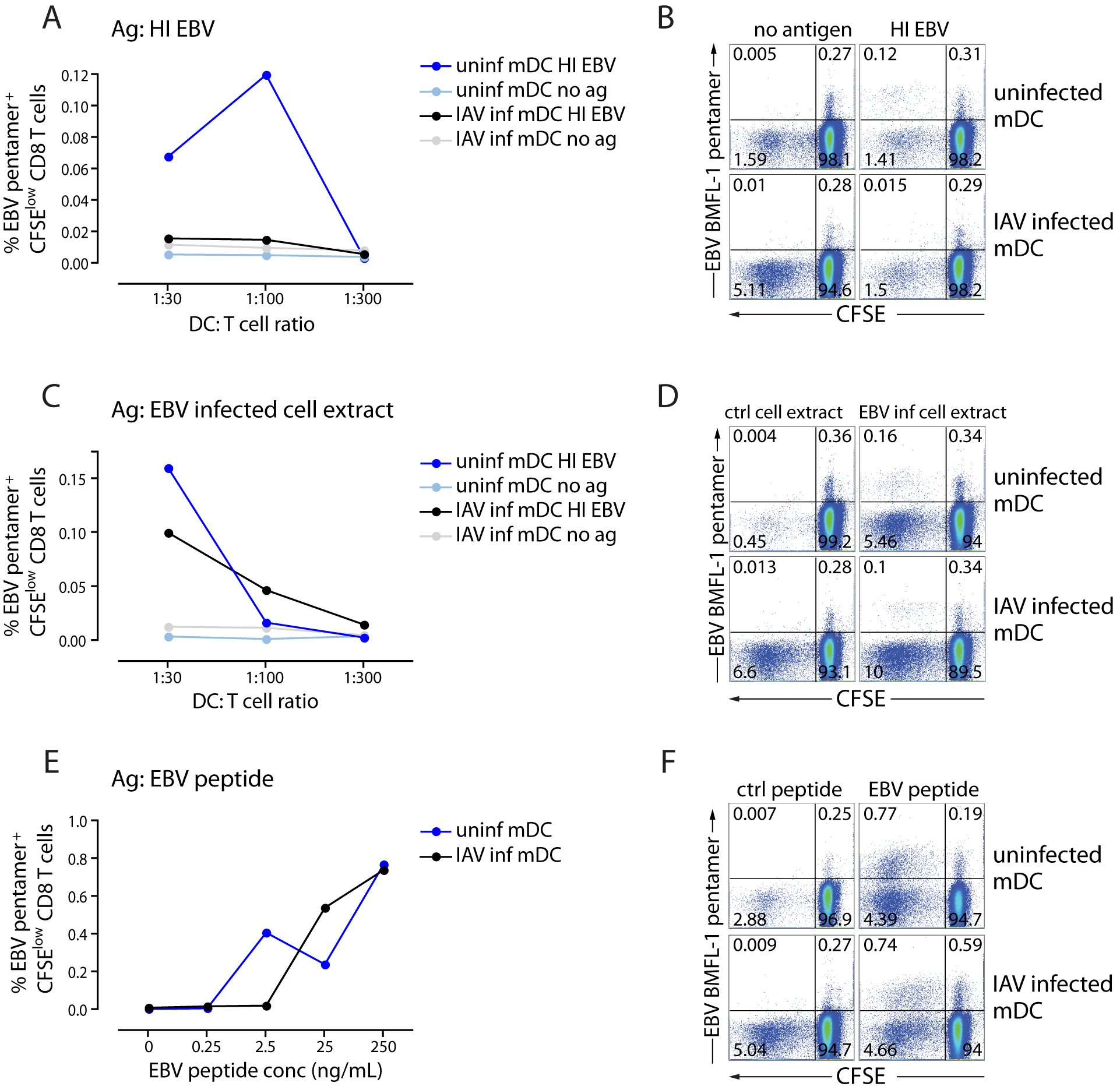

Supplement: Figure S7 — IAV infected mDCs cross-present EBV less efficiently to CD8 T cells than uninfected mDCs. mDCs were infected with infectious IAV or not for 4 hr, washed to remove non-cell associated virus and exposed to (A–B) HI EBV, (C–D) EBV infected or control cell extract, or (E–F) increasing doses of EBV MBLF-1 peptide (GLCTLVAML) for an additional 3 hr. mDCs were then washed and co-cultured with autologous CFSE-labeled CD8 T cells at (A–D) different or (E–F) 1∶30 DC∶T cell ratios. After 10 days of co-culture, cells were harvested and stained with an HLA-A2 EBV BMLF-1 pentamer to detect EBV BMLF-1-specific CD8 T cells and analyzed by flow cytometry. T cell proliferation was detected by CFSE dilution. (A, C, E) The graph shows frequency of EBV pentamer+ CFSElow CD8 T cells after co-culture with uninfected mDCs (blue) or IAV infected mDCs (black). (B–F) Dot plots show live CD8 T cells and numbers indicate frequency of positive CD8 T cells at (B) 1∶100 or (D) 1∶30 DC∶T cell ratio or (F) co-cultured with mDCs loaded with 250 ng/mL EBV peptide. One representative experiment of 3 is shown. (TIF) [file ppat.1002572.s007.tif]
